# Heat-Killed *Lactobacillus salivarius* and *Lactobacillus johnsonii* Reduce Liver Injury Induced by Alcohol In Vitro and In Vivo

**DOI:** 10.3390/molecules21111456

**Published:** 2016-10-31

**Authors:** Cheng-Hung Chuang, Cheng-Chih Tsai, En-Shyh Lin, Chin-Shiu Huang, Yun-Yu Lin, Chuan-Ching Lan, Chun-Chih Huang

**Affiliations:** 1 Department of Nutrition, Master Program of Biomedical Nutrition, Hungkuang University, 1018 Sec. 6 Taiwan Boulevard, Taichung 43302, Taiwan; lin10554@gmail.com; 2 Department of Food Science and Technology, Hungkuang University, Taichung 43302, Taiwan; tsaicc@sunrise.hk.edu.tw; 3 Department of Beauty Science, National Taichung University of Science and Technology, No. 193, Sec. 1, San-Min Rd., Taichung 40343, Taiwan; eslin7620@hotmail.com; 4 Department of Health and Nutrition Biotechnology, Asia University, 500 Lioufeng Rd., Wufeng, Taichung 41354, Taiwan; cshuang@asia.edu.tw; 5 Department of Medical Research, China Medical University Hospital, China Medical University, Taichung 40402, Taiwan; 6 New Bellus Enterprises Co., Ltd. No. 48, Industrial Rd., Erh Chen Vil., Kuan Tien Dist., Tainan 72042, Taiwan; alicelan.nb@gmail.com (C.-C.L.); john@newbellus.com.tw (C.-C.H.)

**Keywords:** alcoholic liver disease, γ-glutamyl transferase, heat-killed *Lactobacillus*, malondialdehyde, *Lactobacillus salivariu*, *Lactobacillus johnsonii*

## Abstract

The aim of the present study was to determine whether *Lactobacillus salivarius* (LS) and *Lactobacillus johnsonii* (LJ) prevent alcoholic liver damage in HepG2 cells and rat models of acute alcohol exposure. In this study, heat-killed LS and LJ were screened from 50 *Lactobacillus* strains induced by 100 mM alcohol in HepG2 cells. The severity of alcoholic liver injury was determined by measuring the levels of aspartate transaminase (AST), alanine transaminase (ALT), gamma-glutamyl transferase (γ-GT), lipid peroxidation, triglyceride (TG) and total cholesterol. Our results indicated that heat-killed LS and LJ reduced AST, ALT, γ-GT and malondialdehyde (MDA) levels and outperformed other bacterial strains in cell line studies. We further evaluated these findings by administering these strains to rats. Only LS was able to reduce serum AST levels, which it did by 26.2%. In addition LS significantly inhibited serum TG levels by 39.2%. However, both strains were unable to inhibit ALT levels. In summary, we demonstrated that heat-killed LS and LJ possess hepatoprotective properties induced by alcohol both in vitro and in vivo.

## 1. Introduction

Excess alcohol intake is one of the causes of chronic liver diseases and can lead to death [1]. The development of alcoholic liver disease (ALD) is complex and involves multiple steps, which include alcoholic steatosis, alcoholic hepatitis, alcoholic cirrhosis and, eventually, liver cancer [2,3]. Alcohol is metabolized by alcohol dehydrogenase, and its by-products may disrupt the normal physiology of liver cells and result in injury. For example, a high concentration of nicotinamide adenine dinucleotide (NAD) hydrate is produced during the process, thus increasing the NADH/NAD^+^ ratio. This enhanced ratio can inhibit lipolysis and decrease the levels of free fatty acids, resulting in triglyceride (TG) accumulation and, subsequently, fatty liver [4,5]. Alcohol also increases the production of oxygen reactive species and oxidative stress in the liver. Oxidative stress can cause mitochondrial damage, protein degradation and inflammation. Individuals who abuse alcohol often have ALD [6,7,8,9]. Antioxidants such as vitamin E and resveratrol have been shown, however, to reduce alcoholic liver damage [10,11].

*Lactobacillus* strains have been classified as Generally Recognized as Safe Foods (GRAS) by the United States Food and Drug Administration (Rockwell, MD, USA). Moreover, many *Lactobacillus* strains have been demonstrated to have anti-oxidant properties [12,13,14,15]. For example, in vitro experiments demonstrated that *Lactobacillus acidophilus* (*L. acidophilus*) ATCC 4356 depletes DPPH free radicals and inhibits the peroxidation of linoleic acid [13]. Clinical studies [16,17,18] and animal studies [19,20,21,22] further demonstrated that both probiotic and prebiotic bacteria improve or prevent acute and chronic diseases, including ALD. Furthermore, research by Kirpich et al. [22] showed that, in alcoholic patients, five days of supplementation with *Bifidobacterium bifidum* and *Lactobacillus plantarum* 8PA3 increased the numbers of fecal bifidobacteria, lactobacilli and enterococci and lowered serum aspartate transaminase (AST) and alanine transaminase (ALT) levels. Among patients with mild hepatitis, probiotic supplementation decreased AST, ALT, gamma-glutamyl transferase (γ-GT), lactate dehydrogenase and total bilirubin levels. In a rat model, feeding strains of *Lactobacillus rhamnosus* Gorbach-Goldin (LGG) decreased toxin formation and alleviated the degree of alcohol-induced liver injury [23]. In addition, LGG has been reported to reduce oxidative stress and inflammation induced by alcohol in both the liver and intestines [24]. 

Hence, *Lactobacillus* strains have shown promising potential for preventing ALD. However, there have not been many studies on the effects of heat-killed *Lactobacillus* strains against ALD. To our knowledge, the only previous study of that sort was by Segawa et al. [25], who reported that the oral administration of heat-killed *Lactobacillus brevis* (*L. brevis*) SBC8803 to C57BL/6N mice ameliorated alcohol-induced liver injury. This hepatoprotective effect was likely due to the suppression of tumor necrosis factor α and the induction of sterol regulatory element-binding protein (SREBP) expression. Additionally, *L. brevis* SBC8803 reduced endotoxin levels in the liver and enhanced intestinal barrier function, thus preventing toxins from entering the liver. In this study, we screened 50 strains of heat-killed *Lactobacillus* and studied their hepatoprotective effects in HepG2 cells. *Lactobacillus salivarius* (LS) and *Lactobacillus johnsonii* (LJ) demonstrated the strongest effects in terms of lowering AST, ALT and γ-GT levels. Therefore, we further investigated the effects of LS and LJ in animal models of acute alcohol exposure.

## 2. Results

### 2.1. Effects of Heat-Killed LS and LJ on AST, ALT and γ-GT Levels in HepG2 Cells

**Figure 1:**
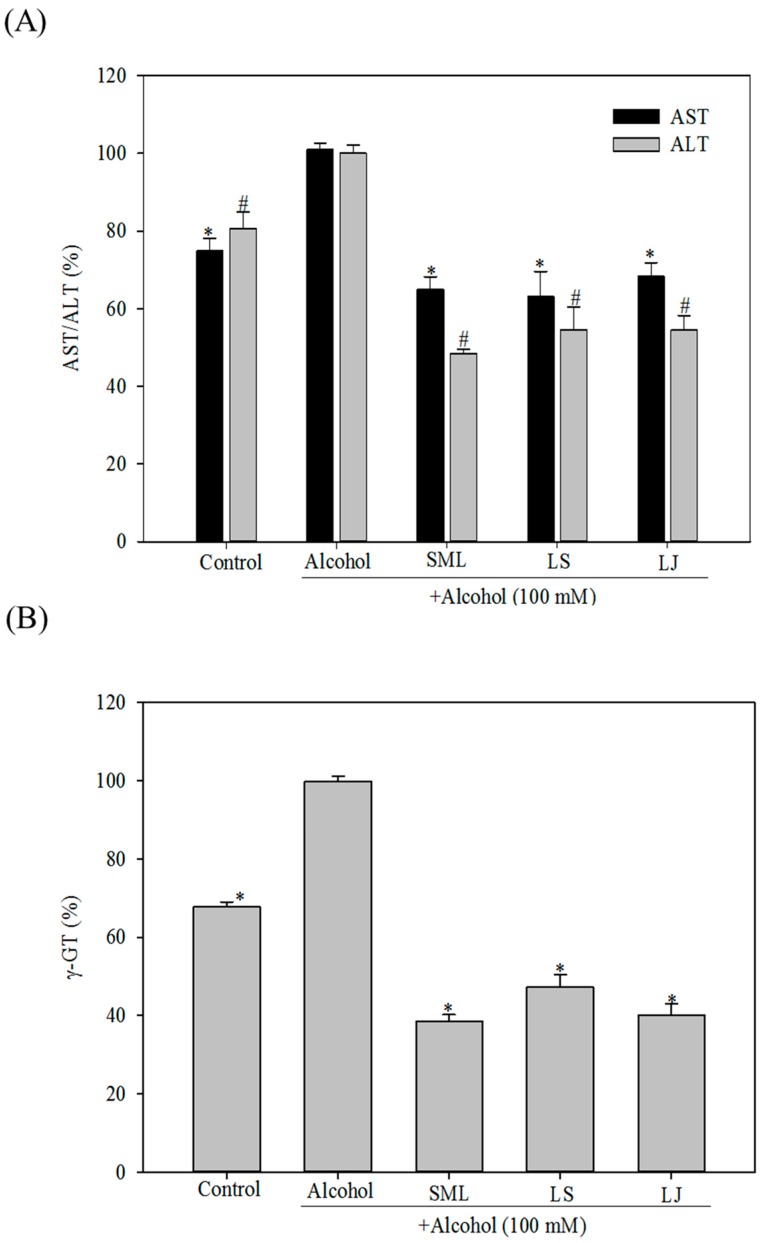
Effects of heat-killed *Lactobacillus salivarius* (LS), *Lactobacillus johnsonii* (LJ) and silymarin (SML) on aspartate transaminase (AST) and alanine transaminase (ALT; (**A**)); and γ-glutamyl transferase (γ-GT; (**B**)) levels in human HepG2 cells induced by alcohol. Experimental conditions were as described in the Materials and Methods. Bars represent mean ± SD (*n* ≥ 3). * *p* < 0.05 or ^#^ *p* < 0.05 for any alcohol treatment groups and control group versus alcohol group.

The degree of liver injury due to alcohol treatment was assessed by measuring the levels of AST, ALT and γ-GT. Silymarin (SML) is a substance extracted from *Silybum marianum* that contains flavonolignans, which are capable of preventing liver injury caused by chemicals and toxic substances such as carbon tetrachloride and alcohol [26,27]. One hundred mg/kg body weight or higher doses of SML have been widely used as a standard hepatoprotective agent [27,28]. Hence, SML served as a positive control in the present study. As shown in Figure 1A, the ASL and ALT levels were significantly increased in the alcohol-treated group compared with the control group (*p* < 0.05). SML treatment significantly reduced the AST and ALT levels by 35.6% and 51.6%, respectively. LS treatment lowered the AST and ALT levels by 47.5% and 55.4%, respectively (*p* < 0.05), whereas LJ treatment lowered the AST and ALT levels by 42.2% and 55.6%, respectively (*p* < 0.05). γ-GT, which is secreted by the liver and gallbladder, is a common biochemical marker for alcoholic liver injury, as the levels of γ-GT become elevated as the result of such injury. SML treatment lowered γ-GT levels by 61.3%, while LS and LJ treatments also significantly reduced γ-GT levels by 52.7% and 59.8%, respectively (Figure 1B).

### 2.2. Effects of Heat-Killed LS and LJ on Lipid Peroxidation in HepG2 Cells

**Figure 2:**
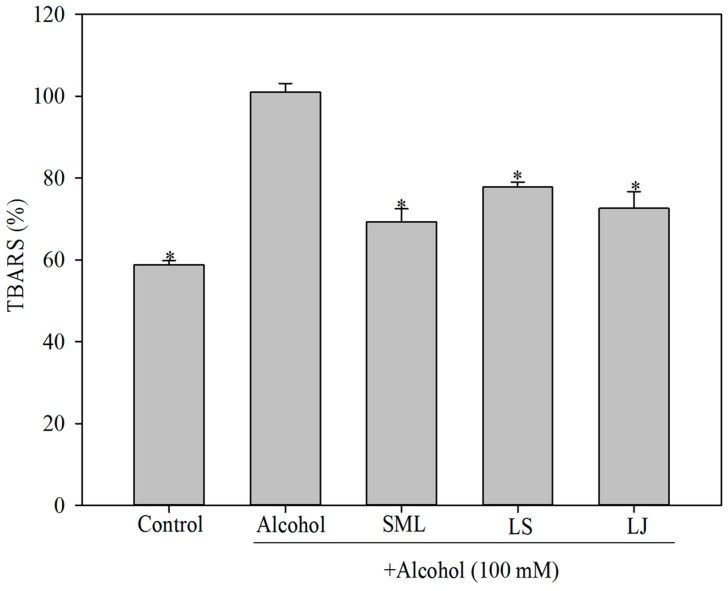
Effects of heat-killed *Lactobacillus salivarius* (LS), *Lactobacillus johnsonii* (LJ) and silymarin (SML) on malondialdehyde (MDA) levels in human HepG2 cells induced by alcohol. Experimental conditions were as described in the Materials and Methods. Bars represent mean ± SD (*n* ≥ 3). * *p* < 0.05 for any alcohol treatment groups and control group versus alcohol group.

Thiobarbituric acid-reactive substances (TBARS) analysis mainly measures the levels of secondary products of lipid peroxidation-malondialdehyde (MDA), which is a common indicator for oxidative stress. MDA was used in the study to investigate the degree of oxidative stress caused by alcohol treatment. As expected, MDA concentrations were significantly higher in the alcohol group as compared with the control group (Figure 2). SML treatment decreased MDA concentrations by 31.4%, while the LS and LJ groups also showed notable reductions in MDA levels of 22.0% and 28.1%, respectively (*p* < 0.05).

### 2.3. Effects of Heat-Killed LS and LJ on γ-GT Levels in Animal Models

**Figure 3:**
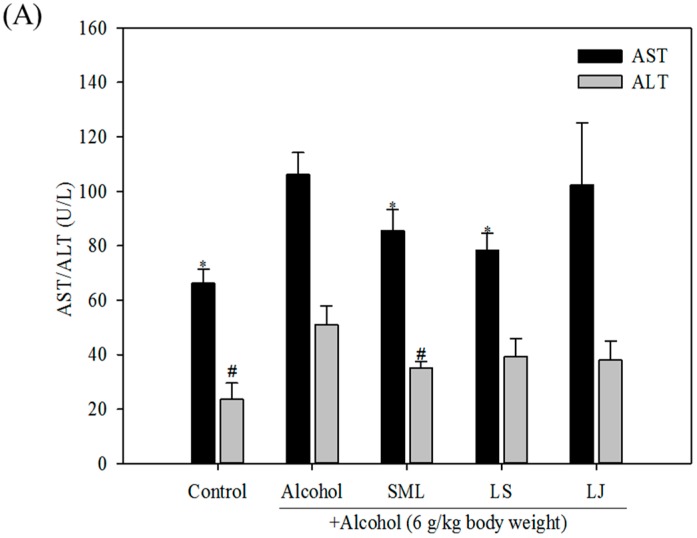

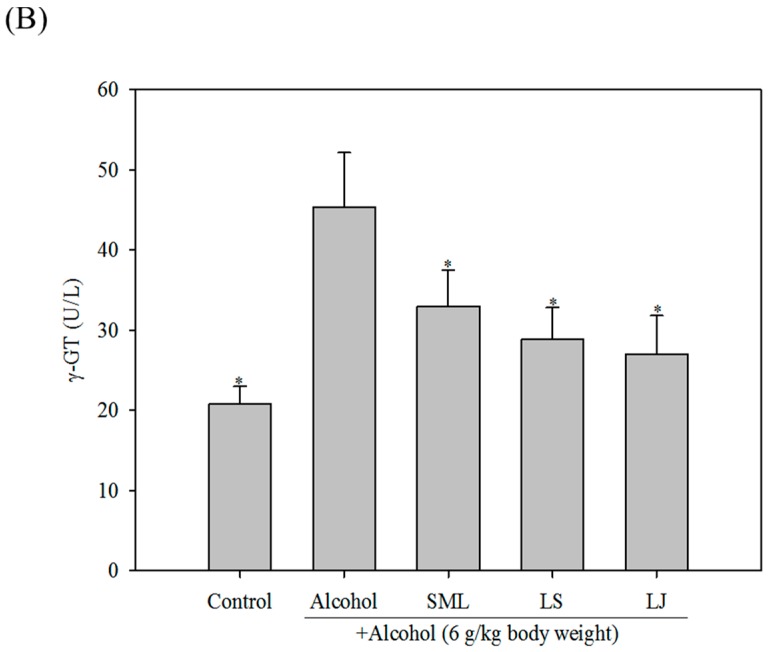
Effects of heat-killed *Lactobacillus salivarius* (LS), *Lactobacillus johnsonii* (LJ) and silymarin (SML) on serum aspartate transaminase (AST) and alanine transaminase (ALT; (**A**)); and γ-glutamyl transferase (γ-GT; (**B**)) levels in rats induced by alcohol. Experimental conditions were as described in the Materials and Methods. Bars represent mean ± SD (*n* ≥ 9). * *p* < 0.05 or ^#^ *p* < 0.05 for any alcohol treatment groups and control group versus alcohol group.

To confirm the above in vitro findings, we investigated the effects of LS and LJ treatments on γ-GT in a rat model of acute alcohol exposure. After 10 days of feeding with *Lactobacillus* strains, the animals had an average body weight of 274.2 g. No significant difference was found among the groups, which indicated that the heat-killed LS and LJ had no effect on the normal growth of the rats. Moreover, no difference in liver and kidney weights was found among the groups (Table S1). Hence, no obvious toxicities were observed based on the above findings. As shown in Figure 3A, the alcohol group had significantly higher levels of AST and ALT. SML treatment resulted in lower AST and ALT levels. Unlike in the in vitro studies, only LS treatment significantly lowered the AST levels, which it did by 26.2%. Meanwhile, no significant effects on the ALT levels were exerted by either the LS or LJ treatment. On the other hand, the LS and LJ treatments more effectively lowered γ-GT levels (by 36.6% and 40.5%, respectively) than the SML treatment (27.3%; Figure 3B).

### 2.4. Effects of Heat-Killed LS and LJ on Lipid Peroxidation in Animal Models

**Figure 4:**
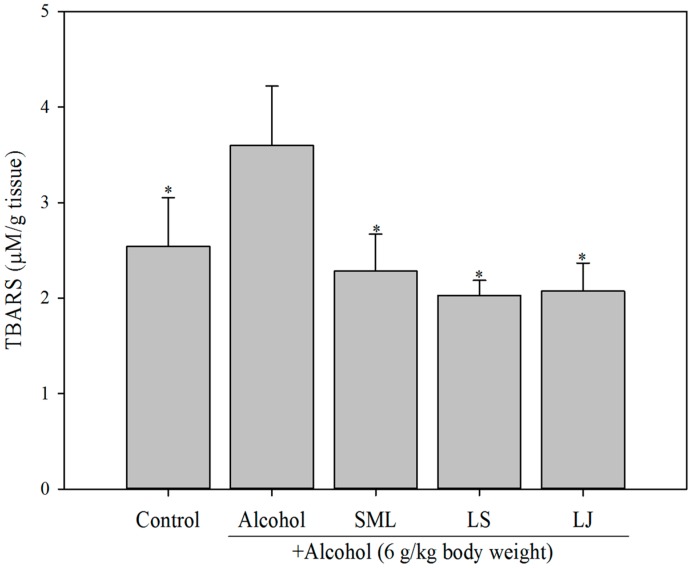
Effects of heat-killed *Lactobacillus salivarius* (LS), *Lactobacillus johnsonii* (LJ) and silymarin (SML) on malondialdehyde (MDA) levels in hepatic tissues of rats induced by alcohol. Experimental conditions were as described in the Materials and Methods. Bars represent mean ± SD (*n* ≥ 9). * *p* < 0.05 for any alcohol treatment groups and control group versus alcohol group.

MDA levels in the alcohol-treated group were elevated as compared with the control group. The SML, LS and LJ treatments all significantly lowered MDA concentrations (by 36.1%, 44.4% and 41.7%, respectively (Figure 4; *p* < 0.05).

### 2.5. Effects of Heat-Killed LS and LJ on Blood Lipid Levels in Animal Models

Excess consumption of alcohol may lead to hypertriglyceridemia and an elevated level of very low density lipoprotein [29]. After feeding with heat-killed LS and LJ for ten consecutive days, followed by alcohol induction (6 g/kg body weight), the SML treatment did not result in reduced TG levels, while the LS treatment significantly lowered TG levels (*p* < 0.05; Table 1). No difference was observed for high density lipoprotein (HDL)-cholesterol, low density lipoprotein (LDL)-cholesterol or total cholesterol levels among the groups. 

## 3. Discussion

Excess alcohol intake is one of the causes of chronic liver disease. In the present study, we showed that heat-killed LS and LJ alleviated the elevations of ALT, AST, γ-GT, MDA and TG in rat models of acute alcohol exposure. First, we screened 50 strains of *Lactobacillus* in HepG2 cells for their hepatoprotective potential. HeG2 cells have been widely used for studying alcohol-induced liver injury [30,31]. When a liver cell is damaged, cellular ALT and AST will leak out into the circulation, resulting in elevated AST and ALT levels in the serum [9]. γ-GT is a cholestatic liver enzyme and largely stored in the liver. When the bile duct is obstructed, γ-GT accumulates and leaks out into the blood stream [32]. Our results showed that heat-killed LS and LJ were able to reduce the elevation of these three enzymes in vitro. In the animal studies, both LS and LJ effectively lowered the γ-GT levels. However, only LS had a significant effect on AST levels, and neither LS nor LJ reduced the ALT levels. These results differed from those of the cell line studies. Such differences are common, however, as in vitro results do not necessarily translate into in vivo results. 

It is generally thought that oxidative stress may contribute to liver toxicities, which further advance to ALD [33]. Studies have found that oxidative stress and lipid peroxidation are closely associated with the formation of fatty liver [33,34]. As MDA is a product of lipid peroxidation, it is commonly used as a biomarker for oxidative stress [35]. When excess alcohol is given to animals, alcohol alters the permeability of the plasma membrane, resulting in enzyme leakage and increased levels of lipid peroxidation products [36]. Lieber [37] demonstrated that unstable permeability due to alcohol insult leads to lipid peroxidation. Other *Lactobacillus* strains have also been confirmed to have antioxidant properties [12,13,14,15]. For example, Lin and Chang [13] demonstrated that both intact cells and intracellular cell-free extracts of *Bifidobacterium longum* ATCC 15708 and *L. acidophilus* ATCC 4356 have the ability to scavenge the 1,1-diphenyl-2-picrylhydrazyl (DPPH) free radical and inhibit the peroxidation of linoleic acid. In addition, in vivo studies showed that *Streptococcus thermophilus* 1131 and *Lactobacillus delbrueckii* subsp. *bulgaricus* 2038 exhibit a high scavenging ability against free radicals and reduce the oxidation of erythrocyte membranes [14]. In this study, both in vitro and in vivo experiments demonstrated that heat-killed LS and LJ may inhibit MDA production. Superoxide dismutase (SOD), catalase and glutathione peroxidase (GPx) are the major antioxidant enzymes in the liver. Past studies have reported that alcoholic liver damage induces the expression of SOD, catalase and GPx [33,38]. Thus, we further tested the effect of heat-killed LS and LJ on SOD, catalase and GPx activities. LS significantly increased the activities of catalase and GPx in liver tissues. In contrast, LJ did not significantly affect the activities of antioxidant enzymes (*p* > 0.05; Figure S1). Therefore, our results suggest that LS decreases MDA levels in liver tissues induced by alcohol, and is at least partially associated with increased activities of catalase and GPx. Further studies are required to elucidate the mechanisms by which LS and LJ exert their antioxidant effects.

The accumulation of TG and cholesterol in the serum and liver is one of the main causes for fatty liver disease. Alcohol consumption increases serum TG levels, and these increased TG levels lead in turn to the fatty liver condition. In previous clinical studies, it has been reported that approximately 80% of heavy alcohol drinkers have the fatty liver condition [1,3]. Our present findings include the finding that LS significantly reduced the levels of serum TG in rats (*p* < 0.05). One of the molecular mechanisms responsible for this involves SREBP, which plays an important role in TG synthesis in the liver [39]. Alcohol uptake enhances SREBP activation. Hence, the overexpression of SREBP-1 causes TG elevation and, eventually, the fatty liver condition [25,40,41]. Segawa et al. [25] found, however, that the oral administration of *L. brevis* SBC8803 in C57BL/6N mice alleviated SREBP expression and lowered TG levels in the liver tissues. Whether LS inhibits serum TG levels by influencing SREBP expression requires further investigation.

## 4. Materials and Methods

### 4.1. Reagents

Bovine serum albumin (BSA) and SML were purchased from Sigma-Aldrich (St. Louis, MO, USA). MRS broth was purchased from Difco Laboratories (Detroit, MI, USA). Dulbecco’s Modified Eagle Medium (DMEM), trypsin, streptomycin, sodium pyruvate and nonessential amino acids were purchased from GIBCO/BRL (Rockville, MD, USA). Fetal bovine serum (FBS) was purchased from Hyclone (Logan, UT, USA). The above reagents were of reagent grade I or molecular biology grade. 

### 4.2. *Lactobacillus* Cultures and Heat-Killed Products

LS and LJ were provided by New Bellus Enterprises Co., Ltd., Tainan, Taiwan. These two strains were incubated for 24 h at 37 °C in 3 mL MRS broth medium containing 0.5% cysteine. The revived culture was then added to 5 mL MRS broth medium and incubated for 24 h at 37 °C. The culture was then enumerated and the suspension adjusted, such that it contained 2 × 10^9^ CFU/mL. One milliliter of the suspension of LS or LJ was autoclaved for 20 min at 121 °C, and samples prepared in this manner served as the experimental samples used in the present study.

### 4.3. Cell Culture and Treatments

HepG2 cell line (human hepatocellular liver carcinoma cell line) was purchased from the Bioresource Collection and Research Center (Hsinchu, Taiwan). HepG2 cells were cultured in DMEM medium containing (10% FBS, 0.37% sodium hydrogen carbonate, 100 U/mL penicillin and 100 U/mL streptomycin) at a density of 1 × 10^5^ cells/mL at 37 °C and 5% CO_2_. When the cells grew to 80% confluence, the medium was changed to DMEM medium (FBS free). Fifty microliters heat-killed suspension, SML (30 µg/mL) and 100 mM alcohol were also added. After 24 h, the cells were washed twice with phosphate-buffered saline. The medium and the cells were then used for analysis. 

### 4.4. Animals and Experimental Design

Six-week old male Spraque-Dawley (140–170 g, *n* = 45) rats were purchased from BioLASCO Taiwan Co., Ltd. (Taipei, Taiwan). The animals were housed individually in hanging wire mesh cages in a temperature (25 ± 2 °C) and humidity (65% ± 5%) controlled room with an alternating 12-h light:dark cycle. Upon arrival, the animals were acclimated for 1 week, during which they were fed a standard rodent diet (Lab 5001; Purina Mills, St. Louis, MO, USA) and water ad libitum. After one week of acclimation, the animals were randomly assigned to five groups of nine rats each. Each group was administered a dose of one of five treatments at respective time points. The groups were: (i) the control group; (ii) the alcohol group; (iii) the SML group; (iv) the LS group; and (v) the LJ group. The animal study was designed according to the methods described by Seth et al. [3]. Alcohol, SML, LS and LJ were mixed with normal saline. During the study, animals in control and alcohol group were orally gavaged with normal saline. The SML, LS and LJ groups were administered SML (200 mg/kg body weight), heat-killed LS (1 × 10^11^ CFU/kg body weight) and heat-killed LJ (1 × 10^11^ CFU/kg body weight), respectively. On Day 10, the control group was given an equal volume of normal saline while the other groups were orally gavaged with a single dose of alcohol (1 mL of 6 g/kg body weight as 30% saline solution). The animals were anesthetized with isoflurane. After sacrifice, blood and liver tissue were collected, snap frozen with liquid nitrogen and stored at −80 °C until analysis. The study protocol was approved by the Animal Research Committee at the Hungkuang University (Taichung, Taiwan, approval #98014).

### 4.5. Measurement of ALT, AST and γ-GT Activity

The extent of alcohol-induced damage was assessed by measuring ALT, AST and γ-GT activity. The activities of ALT, AST and γ-GT were measured by respective commercial assay kits according to the manufacturer’s protocol (Randox laboratories Ltd., Antrim, UK; kits cat. No. AL1268, AS1267 and GT523, respectively).

### 4.6. Measurement of Lipid Peroxidation

The measurements of lipid peroxidation were performed according to Buege and Aust [42] and Imamoglu et al. [43]. Dibutyl hydroxy-toluene was added to 1 mL of cell supernatant or liver homogenate to the final concentration of 0.5 mM. One milliliter of 2.5% trichloroacetic acid and 1 mL 0.7% thiobarbituric acid were also added and the mixture was incubated for 10 min in a 100 °C water bath. After cooling, 3 mL of butanol was added and the solution was centrifuged at 1000× *g* for 5 min. One milliliter of solution was analyzed by the spectrophotometer (Excitation: 515 nm; Emission: 555 nm). MDA from the reaction of 1,1,3,3-tetramethoxypropane and 1 N H_2_SO_4_ was used as standards. The amount of TBARS was expressed as nmol MDA/mg protein.

### 4.7. Measurement of Serum Lipids

Serum total cholesterol, HDL-cholesterol and LDL-cholesterol levels were measured with commercial kits according to the manufacturer’s protocol (Randox laboratories Ltd., Antrim, UK; kits cat. No. TR 213 and CH201; Fortress diagnostics limited, Antrim, UK; kits cat. No. BXC0421E and BXC0431E, respectively).

### 4.8. Determination of Liver Antioxidant Enzymes

The SOD, catalase and GPx activities were each assayed with a commercial assay kit according to the manufacturer’s protocol (Cayman Chemical Co., Ann Arbor, MI, USA; Item No. 706002, 707002 and 703102, respectively).

### 4.9. Statistical Analysis

The data were expressed as mean ± SD. Experimental data were analyzed using the *t*-test (Student’s *t*-test) procedure of SPSS 18. A comparison was made between each group and the alcohol group. In addition, we compared group means by using the one-way factorial analysis of variance (ANOVA) followed by Duncan’s multiple-range test. Differences were considered significant if *p* < 0.05.

## 5. Conclusions

In conclusion, heat-killed LS and LJ effectively protected liver cells from alcohol-induced injury, as evidenced by reductions in AST, ALT and γ-GT activities and decreased MDA content in cell line studies. Furthermore, our results also indicated that both LS and LJ effectively lowered the γ-GT levels induced by alcohol in the animal studies. However, only LS had a significant effect on AST levels, while only LS inhibited serum TG levels. These effects may be related to the alleviation of lipid peroxidation and are at least partially caused by enhancing the activity of catalase and GPx. Further studies are warranted to investigate the antioxidant effects of LJ and LS.

## Figures and Tables

**Table 1:** Effects of heat-killed *Lactobacillus salivarius* (LS), *Lactobacillus johnsonii* (LJ) and silymarin (SML) on serum lipid levels in rats induced by alcohol ^1^.

Group	Serum Lipid (mg/dL)			
	HDL Cholesterol	LDL Cholesterol	Total Cholesterol	Triglyceride
Control	76.8 ± 6.2	95.6 ± 9.4	172.2 ± 13.5	43.4 ± 8.1 ^c^
Alcohol	75.2 ± 5.1	101.1 ± 7.1	175.6 ± 7.8	167.8 ± 27.2 ^a^
SML ^2^	68.6 ± 5.2	98.4 ± 4.3	181.8 ± 5.9	165.5 ± 28.9 ^a^
LJ	69.6 ± 5.5	106.6 ± 10.7	171.8 ± 17.2	129.4 ± 39.8 ^ab^
LS	77.8 ± 7.9	104.5 ± 4.9	173.3 ± 20.0	102.1 ± 36.1 ^b^

^1^ Values are means ± SD, *n* = 9; means in a column without a common letter are significantly different, *p* < 0.05; ^2^ SML (200 mg/kg body weight) served as the positive control.

## Supplementary Materials

The materials are available online at http://www.mdpi.com/1420-3049/21/11/1456/s1.

## Abbreviations

AST: Aspartate transaminase
ALT: Alanine transaminase
γ-GT: Gamma-glutamyl transferase
MDA: Malondialdehyde
LS: *Lactobacillus salivarius*
LJ: *Lactobacillus johnsonii*
TG: Triglycerides
SML: Silymarin
TBARS: Thiobarbituric acid-reactive substances
SOD: Glutathione peroxidase
GPx: Malondialdehyde
SREBP: Sterol regulatory element-binding protein
