# Supplementary material for: Heat-Killed Lactobacillus salivarius and Lactobacillus johnsonii Reduce Liver Injury Induced by Alcohol In Vitro and In Vivo
Source: Molecules. 2016 Oct 31;21(11):1456. doi: 10.3390/molecules21111456 (PMC6274176; doi:10.3390/molecules21111456)
Supplement: Supplementary file 1 [file molecules-21-01456-s001.pdf]

Supplementary Material: Heat-Killed *Lactobacillus salivarius* and *Lactobacillus johnsonii* Reduce Liver Injury Induced by Alcohol In Vitro and In Vivo

Cheng-Hung Chuang, Cheng-Chih Tsai, En-Shyh Lin, Chin-Shiu Huang, Yun-Yu Lin, Chuan-Ching Lan and Chun-Chih Huang

Table S1. Effect of heat-killed *Lactobacillus salivarius* (LS), *Lactobacillus johnsonii* (LJ) and silymarin (SML) on body weight, liver weight, and kidney weight in rats induced by alcohol [1].

Treatment		Initial Body Weight (g)	Final Body Weight (g)	Liver/Body Weight (%)	Kindey/Body Weight (%)	Liver (g)	Kidney (g)
**Control**	94.9 ± 1.8 [a]	294.2 ± 31.6 [a]	2.94 ± 0.68 [ab]	0.80 ± 0.08 [a]	7.6 ± 1.4 [a]	2.1 ± 0.4 [a]	
**Alcohol**	93.0 ± 3.8 [ab]	279.3 ± 24.9 [ab]	3.13 ± 0.26 [ab]	0.80 ± 0.07 [a]	7.8 ± 1.5 [a]	2.0 ± 0.4 [a]	
**SML[2]**	92.9 ± 3.0 [ab]	287.2 ± 28.5 [a]	3.32 ± 0.5 [a]	0.81 ± 0.08 [a]	8.5 ± 2.7 [a]	2.1 ± 0.4 [a]	
**LS**	93.8 ± 4.6 [ab]	276.8 ± 17.0 [ab]	3.27 ± 0.3 [a]	0.82 ± 0.09 [a]	8.0 ± 1.4 [a]	2.0 ± 0.3 [a]	
**LJ**	91.7 ± 2.3 [ab]	261.2 ± 26.9 [ab]	3.43 ± 0.22 [a]	0.87 ± 0.06 [a]	8.1 ± 1.0 [a]	2.1 ± 0.3 [a]	

[1] Values are means ± SD, $n \geq 9$; means in a column without a common letter are significantly different, $p < 0.05$. [2] SML (200 mg/kg body weight) served as the positive control.

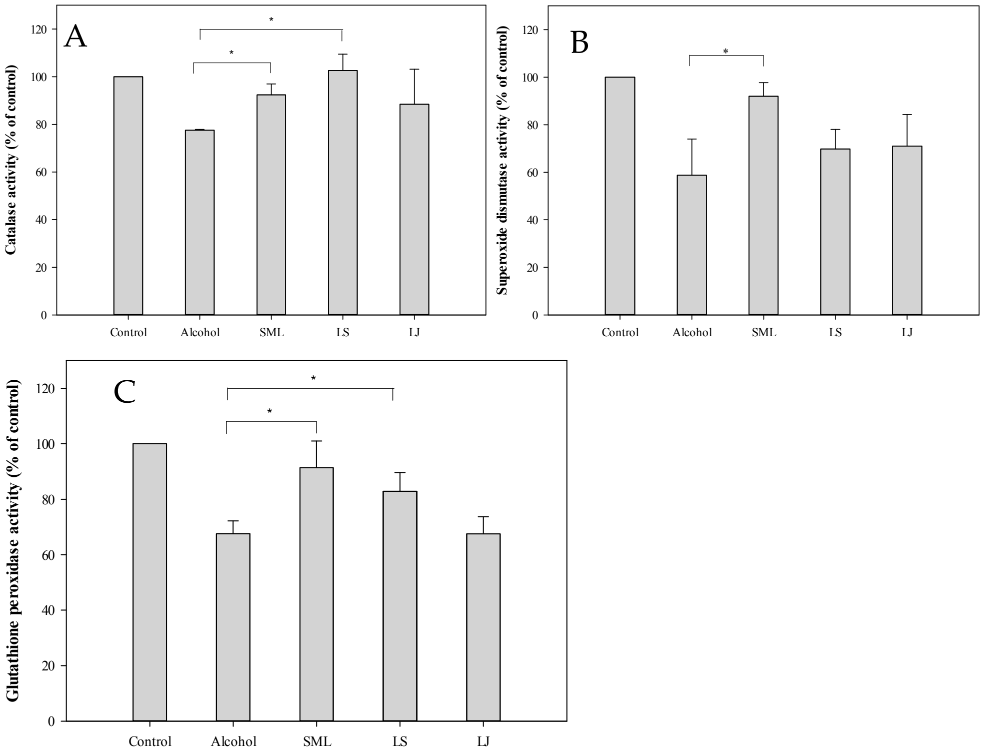

Figure S1. Effects of heat-killed *Lactobacillus salivarius* (LS), *Lactobacillus johnsonii* (LJ) and silymarin (SML) on the activities of superoxide dismutase (**A**), catalase (**B**) and glutathione peroxidase (**C**) in hepatic tissues of rats induced by alcohol. Experimental conditions were as described in Materials and Methods. Bars represent mean ± SD ($n \geq 9$). * $p < 0.05$ for any alcohol treatment groups and control group versus alcohol group.
